# Advances in High-Temperature Non-Metallocene Catalysts for Polyolefin Elastomers

**DOI:** 10.3390/ma18061334

**Published:** 2025-03-18

**Authors:** Cheng Wang, Xin Li, Si Chen, Tianyu Shan

**Affiliations:** 1 College of Chemistry and Chemical Engineering, University of Jinan, Jinan 250024, China; wangcheng02@weixing.com.cn; 2 Satellite Chemical Co., Ltd., Jiaxing 314001, China; lixin02@weixing.com.cn; 3 College of Materials Science & Engineering, Zhejiang University of Technology, Hangzhou 310014, China; chensi@zjut.edu.cn; 4 Department of Chemistry, Stoddart Institute of Molecular Science, Zhejiang University, Hangzhou 310058, China

**Keywords:** non-metallocene catalysts, polyolefin elastomer, thermostability, polyolefin, solution polymerization

## Abstract

Despite the great successes achieved by metallocene catalysts in high-value-added polyolefin elastomer, the challenging preparation conditions and undesirable high-temperature molecular weight capabilities have compromised the efficiency and cost of polyolefin in industrial production. Recently, non-metallocene catalysts have received considerable attention due to their high thermostability, especially when coordinated with early transition metals. This review provides an overview of these early transition metal non-metallocene catalysts, which are mainly composed of *N*,*N*′-, *N*,*O*-, and *N*,*S*-bidentate complexes and tridentate complexes. The structural characteristics, catalytic performance, advantages, and disadvantages of the relevant non-metallocene catalysts, as well as their applications, are discussed. Candidates for commercialization of non-metallocene catalysts are proposed—focusing on imine-enamine, amino-quinoline, and pyridine-imine catalysts—by comparing the successful industrialization cases of metallocene catalysts. Finally, the trend in the research on non-metallocene catalysts and the strategies to address the challenges limiting their commercialization are considered.

## 1. Introduction

Polyolefin elastomers (POEs), an important subset of polyolefin materials, have attracted substantial interest due to the synergistic integration of mechanical robustness, fracture resistance, optical clarity, and thermal endurance [1,2,3]. These materials are structurally characterized by a biphasic architecture that includes crystalline polyethylene “hard” segments and amorphous α-olefin copolymer “soft” domains, which establish reversible physical crosslinking networks essential for elastic recovery. Due to these performance advantages, POEs have a wide range of applications in the automotive industry, energy storage systems, and advanced packaging technologies [4,5,6,7,8]. In industrial applications, POEs are mainly synthesized through solution polymerization, which offers significant advantages in mass and heat transfer [9,10,11,12,13,14,15,16]. However, the high-temperature requirements of industrial production (typically exceeding 120 °C) tend to cause catalyst deactivation [17,18,19]. This limitation underscores the critical necessity of developing thermally stable polyolefin catalysts.

**Figure 1:**
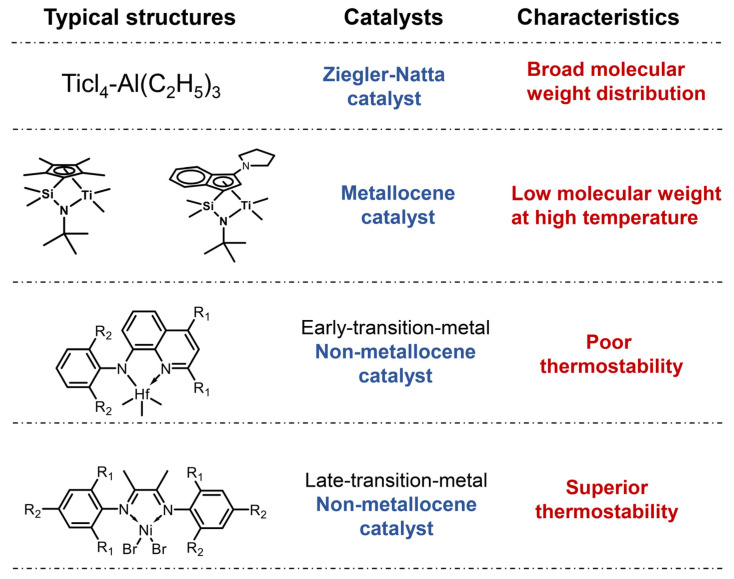
Typical structures of the three types of catalysts and their characteristics.

Over the past half-century, the evolution of polymerization catalysts for POEs—progressing from Ziegler–Natta (Z-N) catalysts to metallocene catalysts and subsequently non-metallocene systems—has evolved into a research focus (Figure 1) [20,21,22]. Nevertheless, after seven decades of development, Z-N catalysts have become progressively inadequate for modern high-performance polyolefin production due to their inherent multisite characteristics [23]. A paradigm shift occurred in 1980 when Kaminsky and Sinn revolutionized polyolefin catalysis by integrating methylaluminoxane (MAO) with dichlorodicyclopentadienyl zirconium (Cp_2_ZrCl_2_), thereby marking the advent of second-generation metallocene catalysts [24]. These single-site catalytic systems demonstrate superior activity, expanded monomer compatibility, and precise stereochemical control, enabling the synthesis of copolymers with narrow molecular weight distributions and uniform comonomer incorporation [25,26,27,28,29,30]. Notably, constrained geometry catalysts (CGCs), co-developed by Exxon and Dow Chemical, achieve exceptional activity exceeding 10^8^ g(polymer)·mol^−1^·h^−1^ under optimized conditions [31,32]. Furthermore, the tunable steric hindrance and electronic effects of metallocene catalysts further permit precise modulation of polyolefin architectures. Despite these advancements, its industrial applications still remain constrained by thermally induced molecular weight depression at elevated temperatures [33,34,35,36].

Following metallocene catalysts, polyolefin catalysts without cyclopentadienyl (Cp) are referred to as non-metallocene catalysts and feature heteroatom ligands coordinated with transition metals [37,38,39,40]. These catalysts maintain the fundamental merits of metallocene catalysts—including single-site characteristics and broad monomer adaptability—with generous ligand synthesis requirements [41,42,43,44,45]. These catalysts could be categorized into early and late transition metal catalysts based on their central metal. Notably, late transition metal non-metallocene catalysts exhibit poor thermal stability (below 120 °C) and are primarily utilized for ethylene copolymerization with polar monomers [46,47,48,49,50]. In contrast, the coordination of non-metallocene compounds with Group IVB metals facilitates covalent bond formation, significantly enhancing thermal stability and rendering them suitable for high-temperature solution polymerization. This review focuses on early transition metal non-metallocene catalysts for high-temperature solution polymerization, examining the recent advances in olefin polymerization according to the number of coordinating atoms in catalysts. Then, we provide perspectives on possible research directions for early transition metal non-metallocene catalysts.

## 2. *N*,*N*-Bidentate Ligands

The *N*,*N*-dentate ligand is one of the most common non-metallocene ligands derived from diamine catalysts [51]. The diimine catalysts exhibited a “chain-walking” growth mechanism, resulting in highly branched polymers. Furthermore, the β-H elimination rate at the metal center was dramatically influenced by the steric shielding effect in the axial space, which could be utilized to prepare ethylene-based polyolefin elastomers (EPOEs) [52,53,54,55,56,57]. These ligands mainly coordinated with late transition metals for copolymerization of ethylene and polar α-olefins, which have been reviewed [46,47,48,49]. To further enhance the thermal stability, ligands including imino-amido, imino-enamido, and amido-quinoline ligands were derived by structural design, which can be further coordinated with an early transition metal for high-temperature polymerization.

### 2.1. Imino-amido Ligands

**Figure 2:**
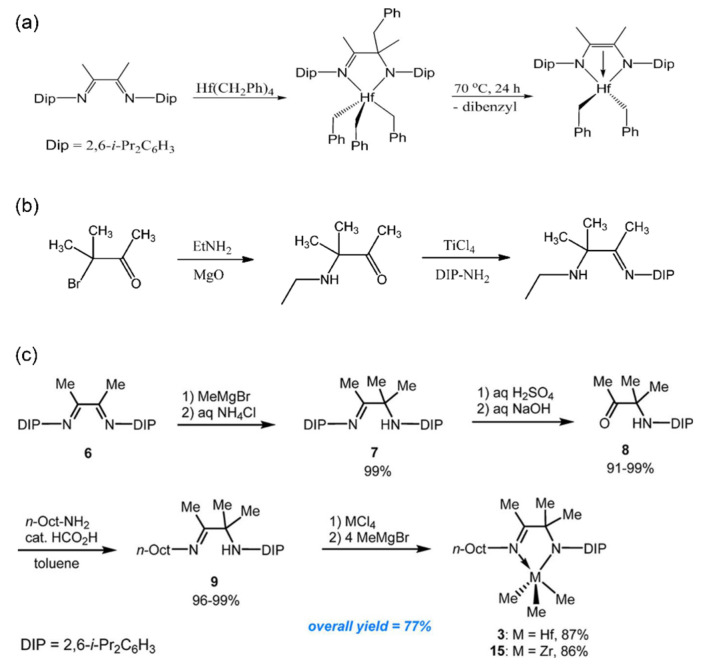
(**a**) Thermal decomposition procedure of the symmetric imine-amino catalyst [58]; (**b**) synthesis route of the asymmetric imine-amino ligand [59]; (**c**) modified synthesis route of the asymmetric imine-amino ligand for scale-up production [60]. Reprinted with permission of American chemical society publication, copyright (2025).

Based on diimine structures, Dow have developed a new family of polyolefin catalysts [58]. They found that alkyl transfer occurred between diimine ligands and transition metal alkyl precursors. Under high polymerization temperature, thermal decomposition with benzyl elimination occurred, forming an ene-diamine structure instead of a second benzyl transfer (Figure 2a). Since the imine-amino Hf complexes exhibit stronger catalytic activity for ethylene and 1-octene copolymerization compared to the ene-diamine Hf complexes, this high-temperature conformational transformation compromises their thermal stability. As a result, it demonstrated the moderate activity of 6.6 × 10^5^ g(PE)·mol^−1^·h^−1^ at 120 °C. Furthermore, the corresponding Zr complexes, which are more prone to ene-diamine conversion, exhibited poorer thermostability and could not be applied at 120 °C.

Subsequently, Froese et al. developed asymmetric imine-amino ligands for POEs (Figure 2b) [59]. The Hf complex demonstrated high copolymerization ability of 1.4 × 10^8^ g(polymer)·mol^−1^·h^−1^ at 120 °C, with a tendency to increase molecular weight rather than inserting 1-octene. These asymmetric complexes underwent methyl rearrangement at 80.4 °C, resulting in a great reduction in catalytic activity. To further explore the potential of Hf complexes for large-scale production, two methylation methods were proposed by Dow: one involves first synthesizing the MMe_4_ intermediates, which are then immediately coordinated with the ligand; the other directly coordinates with MCl_4_ and subsequently methylates using Grignard reagents (MeMgBr, MeMgI) (Figure 2c) [60]. The first method requires quick reaction at low temperatures due to the instability of the MMe_4_ intermediates, which can slowly release methane even at −15 °C. In contrast, the second one involves first preparing a stable -Cl complex followed by methylation, avoiding the low-temperature reaction and facilitating the scale-up production with an overall yield of 77%.

### 2.2. Imino-enamido Ligand

**Figure 3:**
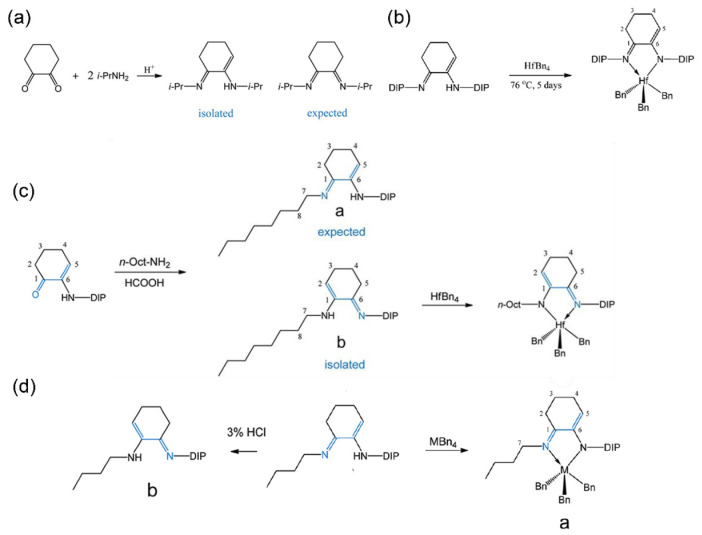
Synthesis routes of (**a**) the imino-enamines ligand, (**b**) Hf complex, (**c**) asymmetric type Hf complex, (**d**) asymmetric type complex [61]. Reprinted with permission of American chemical society publication, copyright (2025).

To prevent the high-temperature isomerization of imine-amino complexes and further enhance their high-temperature polymerization performance, Dow synthesized diimines by condensing 1,2-cyclohexanedione [61]. However, the resulting product was an imino-enamine rather than the expected diimine (Figure 3a). Through computational studies, it was revealed that five- and six-membered rings are more stable in the imino-enamine form, whereas four-, seven-, and eight-membered rings tend to form diimines. To validate the catalytic performance, a benzyl Hf complex of the six-membered-ring imino-enamine ligand was synthesized (Figure 3b). The Hf catalyst demonstrated excellent catalytic properties with a high activity of 2.7 × 10^7^ g(polymer)·mol^−1^·h^−1^, a moderate insertion rate of 5.4 mol%, and a high molecular weight of 10^6^ g·mol^−1^.

Building upon the superiority of asymmetric imine-amino ligands over their symmetric counterparts, the Dow team subsequently synthesized asymmetric imino-enamine ligands. However, the resulting product was the less effective isomer **b** rather than the anticipated isomer **a** (Figure 3c). Then, shortening the alkyl chain length was found to lower the energy barrier between the two isomers. For example, using a butyl chain facilitated the formation of the **a**-type complexes and the improvement of thermostability (Figure 3d). The **a**-type Hf complex exhibited exceptional catalytic performance at 120 °C, with a catalytic activity of 1.2 × 10^8^ g(polymer)·mol^−1^·h^−1^ and a 1-octene insertion rate of 8.7 mol%. Moreover, the catalysts retained both high activity and molecular weight even at 150 °C, a performance level beyond that of CGCs. This groundbreaking discovery makes this **a**-type Hf complex one of the most significant advancements in polyolefin catalysts since CGCs.

**Figure 4:**
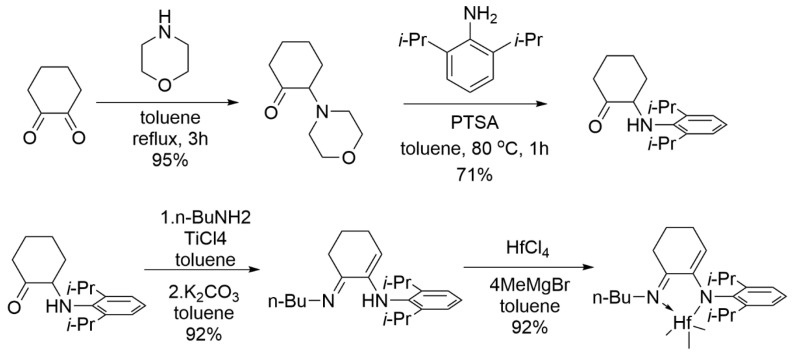
Synthesis route of the imino-enamine Hf complex for scale-up production [62]. Reprinted with permission of American chemical society publication, copyright (2025).

For large-scale production, alternative approaches to replace the expensive HfBn_3_ above have also been proposed (Figure 4) [62]. In a similar manner to the methylation of imine-amino-Hf complexes, imino-enamine ligands can react with HfCl_4_ and MeMgBr to yield the corresponding HfMe_3_ complexes, with a comparable copolymerization efficiency to HfBn_3_. Then, Klosin et al. further explored related derivatizations by using different heteroatom-containing ligands, and only NPtBu_3_ showed improved performance for both catalytic activity and molecular weight by sacrificing the insertion rate [63]. Other analogues with modifications to the enamine skeleton, such as electronically delocalized aminotroponiminato Hf and Zr complexes, failed to deliver satisfactory results [64]. Compared to imine-amino complexes, imino-enamine complexes demonstrate comprehensive improvements in thermostability, catalytic activity, molecular weight, and insertion rates, making them highly valuable for producing POEs.

### 2.3. Amido-quinoline Ligand

**Figure 5:**
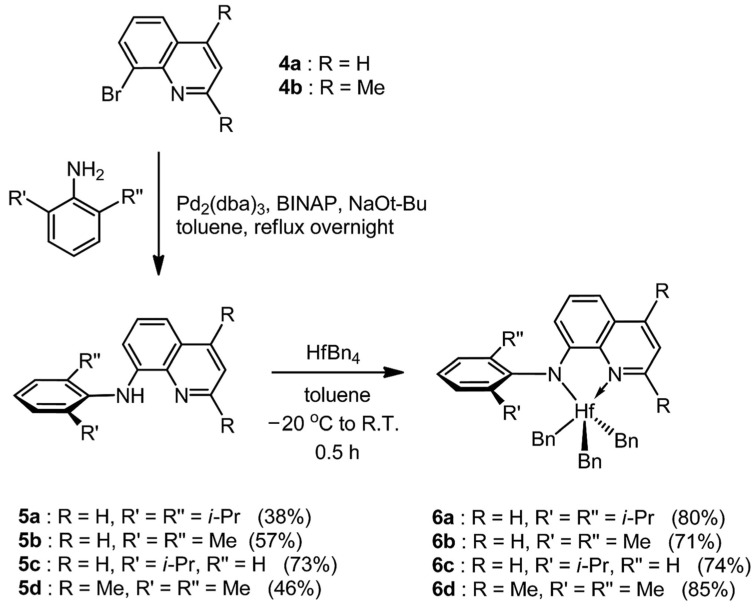
Synthesis routes of the amido-quinoline Hf complexes [65]. Reprinted with permission of American chemical society publication, copyright (2025).

Although imino-enamine ligands present enhanced thermal stability, their rearrangements under acidic conditions remain unavoidable. This presents challenges in both POE preparation and storage, ultimately limiting their commercial viability. To address this issue, a new amido-quinoline catalyst was synthesized in a single step via Pd-catalyzed coupling of 8-bromoquinoline and aniline compounds (Figure 5) [65]. Due to the poor solubility of quinoline derivations in aliphatic hydrocarbons, complexes with amido-quinoline ligands can easily crystallize in toluene/hexane mixtures, thus simplifying the purification. Then, the catalytic performance of quinoline-amino Hf complexes and the substituent effects was carefully investigated. For substituents on the ortho-position of the aniline group, the activity was confirmed by **Me** > **^i^Pr** > **H**. In the absence of ortho-substituents, the metal center becomes more susceptible to attack, leading to β-H elimination or chain transfer. Additionally, the ortho-hydrogen atom on the quinoline nitrogen is abstracted, causing chain transfers and multicenter characteristics (polymer dispersity index (PDI) > 3, when R = H). Conversely, introducing a methyl group at this position narrows the molecular weight distribution without significantly affecting catalytic performance. Compared to the previously discussed imine-amino and imino-enamine complexes, quinoline-amino Hf complexes exhibit comparable activity, insertion rates, and molecular weight (Table 1) and superiority in terms of stability and preparation.

**Figure 6:**
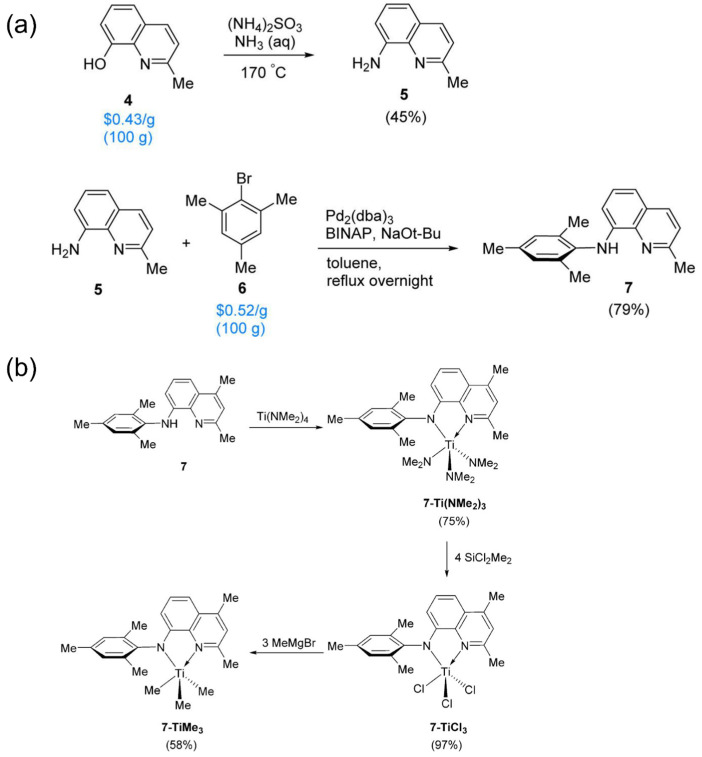
(**a**) High-efficiency synthesis route of the amido-quinoline ligand; (**b**) synthetic route of the related Ti catalyst [67]. Reprinted with permission of American chemical society publication, copyright (2025).

Building on the above successful discovery, Fontaine et al. further optimized the synthesis route of the quinoline-amino Hf complexes to facilitate the real applications [67]. They developed a high-efficiency method to avoid the use of expensive starting material, in which readily available 8-hydroxyquinoline was converted into 8-aminoquinoline, then coupled with 2-bromomesitylene (Figure 6a). And the corresponding Zr and Hf methyl derivatives were successfully synthesized using the same methylation method. For Ti methyl derivatives, the authors also provided an alternative synthetic route (Figure 6b), since TiCl_4_ was sensitive to active hydrogens. By copolymerization experiments of ethylene/1-octene POEs at 140 °C, the Hf complexes were determined to be superior.

**Figure 7:**
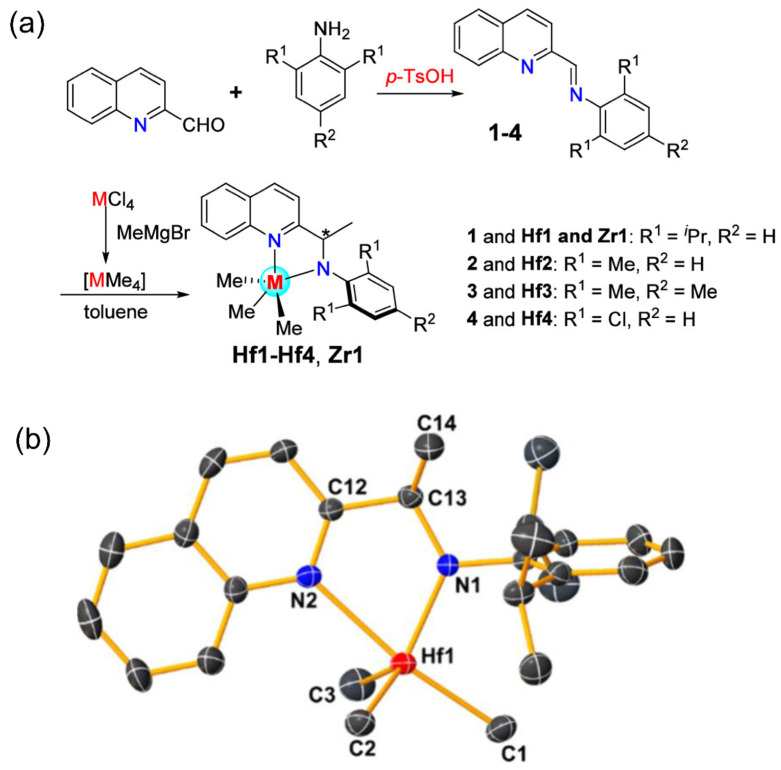
(**a**) Simplified synthesis route of the amido-quinoline complex (the star represents methyl transfer position); (**b**) crystal structure of the Hf catalyst [69]. Reprinted with permission of American chemical society publication, copyright (2025).

Subsequently, Han et al. recently synthesized and studied complexes bearing amido-trihydroquinoline ligands [68]. Although the reduced ring rigidity in amido-trihydroquinoline ligands slightly compromised the thermal stability, it led to a great improvement in 1-octene insertion rates, reaching up to 43.5 mol%. Notably, Zhang et al. designed a quinoline-imine that further simplified the synthesis by avoiding the use of expensive Pd complexes, thereby reducing the costs (Figure 7a) [69]. And when this ligand was complexed by the classical methylation method, the resulting product exhibited an amido-quinoline structure, as confirmed by single-crystal diffraction experiments (Figure 7b). The new amido-quinoline complex avoids ortho effects on the quinoline nitrogen, rendering copolymers with narrow molecular weight distributions. Consequently, the substituent effect on the aniline ortho-position became more significant. In terms of activity, copolymer molecular weight, and 1-octene insertion rates, **^i^Pr** consistently outperformed **Me**, highlighting the steric effect. While substituents in the para-position had minimal impact, **Me** showed slightly better performance than **H**, possibly due to its electron-donating inductive effect. In all cases, the Hf complexes exhibited superior performance to the Zr complexes. Specifically, the quinoline-amino Hf complex demonstrated outstanding catalytic properties at 120 °C, with a high activity of 7.8 × 10^7^ g(polymer)·mol^−1^·h^−1^ and a remarkable 1-octene insertion rate of 49.6 mol%. These features make the quinoline-amino Hf complex particularly promising for the industrial-scale production of POEs. Furthermore, recent studies focused on tridentate ligands based on a quinoline-amino unit, representing another promising avenue. A related detailed summary will be presented in Section 4.

## 3. *N*,*O* and *N*,*S*-Bidentate Ligands

**Figure 8:**
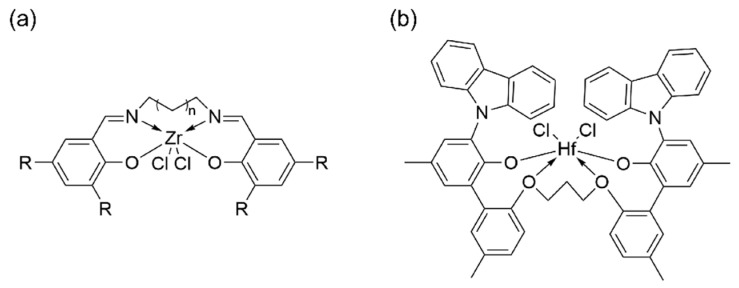
The chemical structures of (**a**) hexamethylene bridge FI catalyst and (**b**) O_4_ catalyst. copyright.

*N*,*O/S*-Bidentate ligands, which mainly consist of phenoxy-imine or imine-ether, generally demonstrate relatively low activity compared to the *N*,*N*-bidentate counterparts. Only limited systems achieve promising performance. Originally, the phenoxy-imine ligands were coordinated with late transition metals, as reported by Grubbs [70]. Leveraging the oxygen tolerance of late transition metals, this system enabled the copolymerization of ethylene with polar monomers. Subsequently, Fujita and Coates independently combined two phenoxy-imine ligands with early transition metals of Zr and Ti to catalyze polyethylene and ethylene–propylene copolymers, known as FI catalysts [71,72]. Though FI catalysts present comparable catalytic activities to metallocene catalysts, their thermal stability is typically poor. Specifically, these FI catalysts are usually applied at 25 °C, and are quickly deactivated at temperatures exceeding 50 °C. To improve the thermal stability of FI catalysts, one of the most important approaches involves the preparation of bridged bis-phenoxy-imine complexes. For example, FI_2_ZrCl_2_, featuring a hexamethylene bridge, exhibited a high activity of 1.43 × 10^8^ g(polymer)·mol^−1^·h^−1^ at 150 °C (Figure 8a) [73]. Related symmetrical bridged structures were first proposed by the Symyx Corporation, in which diphenols were bridged with methylene or other alkane chains to form a tetradentate ligand structure, featuring four oxygen coordination sites (O_4_) (Figure 8b) [74]. These complexes demonstrated excellent high-temperature activity, high molecular weight capability, and considerable comonomer insertion rates. Nevertheless, the above bridged structures are limited by complex synthesis.

**Figure 9:**
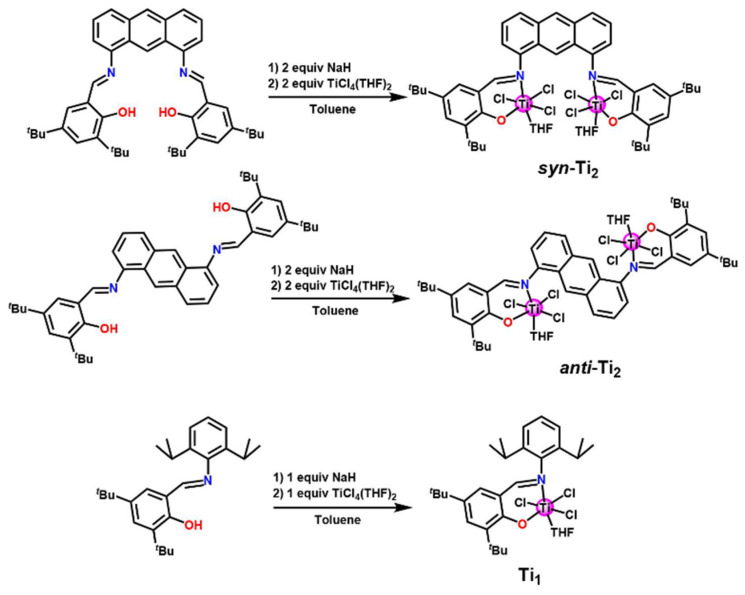
Synthesis route of the anthracene bridged phenoxy-imine Ti catalyst [78]. Reprinted with permission of American chemical society publication, copyright (2025).

Compared to bridged structures, more research has focused on synthesizing phenoxy-imine bimetallic catalysts for their easier preparation [66,75,76,77]. This approach mimicked the cooperative effect and enhanced the metal–metal synergy, making the catalyst thermostable. Liu et al. constructed phenoxy-imine Ti complexes bridged by an anthracene group [78]. Due to the planar nature of the anthracene bridge, the resulting catalysts existed in two different configurations: *syn*-Ti and *anti*-Ti (Figure 9). Homopolymerization experiments confirmed that the *syn*-Ti configuration exhibited superior catalytic performance of 8.6 × 10^4^ g(PE)·mol^−1^·h^−1^ at 100 °C. Subsequently, the team further investigated the high-temperature catalytic performance of anthracene-bridged amino-ether Ti, Zr, and Hf complexes [79]. Consistent with their previous findings, the *syn*-M complexes exhibited superior catalytic performance to their anti-M counterparts. Among them, *syn*-Zr demonstrated exceptional performance in ethylene/1-octene copolymerization at 130 °C, with an activity of 9.6 × 10^6^ g(Polymer)·mol^−1^·h^−1^, a polymer molecular weight of 1.34 × 10^5^ g·mol^−1^ and a 1-octene insertion rate of 2.1 mol%. Notably, through metal–metal synergy, the high-temperature performance is enhanced while sacrificing the single-active-center characteristic.

**Figure 10:**
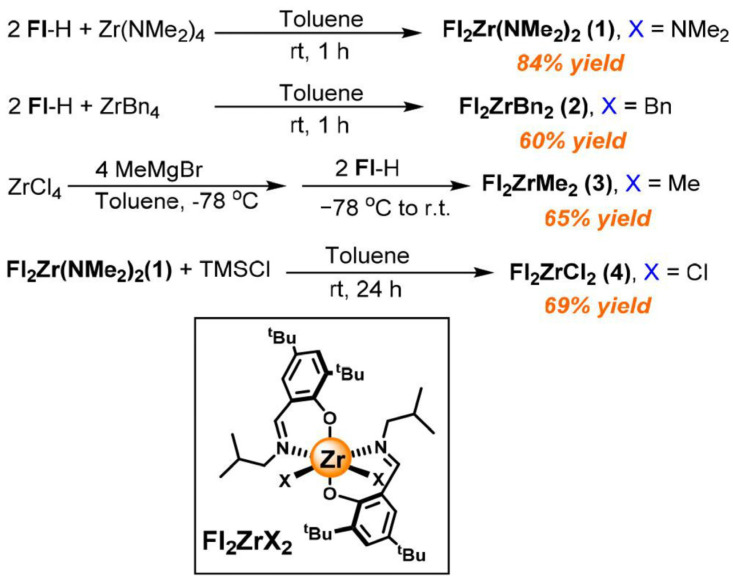
Synthesis routes of the Zr catalysts [80]. Reprinted with permission of American chemical society publication, copyright (2025).

Furthermore, the high-temperature catalyst performance of the above *syn*-Ti catalyst can also be improved by modifying metal–ligand derivatives. Due to the low alkylation efficiency, NMe_2_ ligands can be used as intermediates for the preparation of halogen or alkyl ligand derivatives. For example, Gao et al. synthesized FI_2_Zr(NMe_2_)_2_ complexes, with an activity of 2.3 × 10^6^ g(polymer)·mol^−1^·h^−1^ and a 1-octene insertion rate of 10 mol% at 120 °C, after 30 min of pre-activation with trimethylaluminum (TMA) (Figure 10) [80]. As a contrast, the bridged or bimetallic phenoxy-imine catalysts discussed above face the contradiction between activity and the insertion rate.

**Figure 11:**
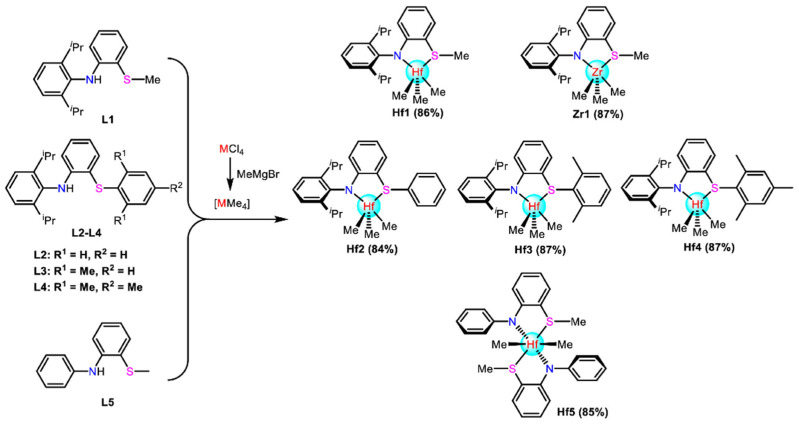
Synthesis routes of the thioether-amino catalysts [84]. Reprinted with permission of American chemical society publication, copyright (2025).

Recently, the combination of hard N-donor and soft S/P-donor ligands was found to demonstrate an unexpected catalytic performance [81,82,83]. The related Ti, Zr, and Hf catalysts were proposed by Liu et al. (Figure 11) [84]. Polymerization experiments demonstrated that the metal center played a critical role in this system: the Hf complexes were superior to the Zr complexes, while the Ti complexes were deactivated. Specifically, the Hf complexes with methyl-substituted thioether ligands demonstrated both a high activity of 2.1 × 10^7^ g(polymer)·mol^−1^·h^−1^ and a high comonomer insertion rate of 34.1 mol% at 120 °C. Although the strategy of combining soft and hard donors was more commonly used in tridentate ligands, the high insertion rate in bidentate ligands offers a unique advantage. The aspects of tridentate ligands are discussed in detail in the next section.

## 4. Tridentate Ligands

Compared to bidentate ligands, tridentate ligands associated with more coordinate or covalent bonds significantly enhance the thermostability. Up to now, numerous tridentate ligands have been reported based on the synergy of hard N donors and soft S/P donors or the combining of *N*,*N*-bidentate ligands and phenoxyimine ligands. However, the increased coordination sites in tridentate ligands lead to greater steric hindrance around the metal center, making the insertion of comonomers more challenging. As a result, linear low-density polyethylene (LLDPE) was typically obtained. Nevertheless, such tridentate catalysts still hold industrial value for their excellent thermostability.

### 4.1. Phenoxy-imine-amino Ligands

**Figure 12:**
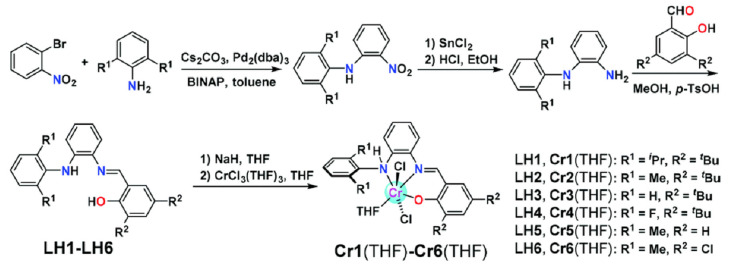
Synthesis routes of the phenoxyimine-amino catalysts [85]. Reprinted with permission of American chemical society publication, copyright (2025).

Tian et al. synthesized phenoxyimine-amino tridentate ligands by cross-coupling, reduction, and condensation reactions, using 1-bromo-2-nitrobenzene as the starting material [85]. The corresponding aniline and salicylaldehyde derivations were subsequently coordinated with CrCl_3_(THF)_3_ to yield phenoxyimine-amino Cr complexes with high yields (Figure 12). By ethylene homopolymerization experiments, these complexes were found to be effectively activated with a slight cocatalyst MAO (Al/Cr = 200), achieving a high catalytic activity of 1.18 × 10^8^ g(PE)·mol^−1^·h^−1^ at 80 °C and retaining a moderate activity of 6.75 × 10^7^ g(PE)·mol^−1^·h^−1^ at 120 °C.

Subsequently, the Tian team further developed the phenoxy-imine-amino Ti complex, demonstrating a high activity of 6.60 × 10^7^ g(PE)·mol^−1^·h^−1^ at 130 °C [86]. Gel permeation chromatography (GPC) confirmed that the molecular weight of the resulting polyethylene was 4 × 10^5^ g·mol^−1^, with a relatively narrow distribution. However, as the temperature increased to 150 °C, the molecular weight distribution widened to 3.31, indicating its multicenter characteristics caused by partial decomposition. Further copolymerization investigation revealed that the insertion of 1-octene effectively suppressed chain transfer at the active center. As a result, the polymerization activity decreased by one order of magnitude compared to homopolymerization, while the molecular weight increased by one order of magnitude.

Regarding phenoxy-imine-amino Zr/Hf complexes, their catalytic performances were recently investigated by Gao et al. [87]. The results revealed that the Zr complex exhibited superior performance to the Hf complex, with a high activity of 9.9 × 10^7^ g(PE)·mol^−1^·h^−1^ at 130 °C. However, the insertion rate of the Zr complex was limited and was determined as 0.29 mol% for 1-octene comonomers. These findings underscore the critical role of the metal center in determining the catalytic behavior of phenoxyimine-amino tridentate ligands.

### 4.2. Phenoxy-imine-quinoline Ligands

**Figure 13:**
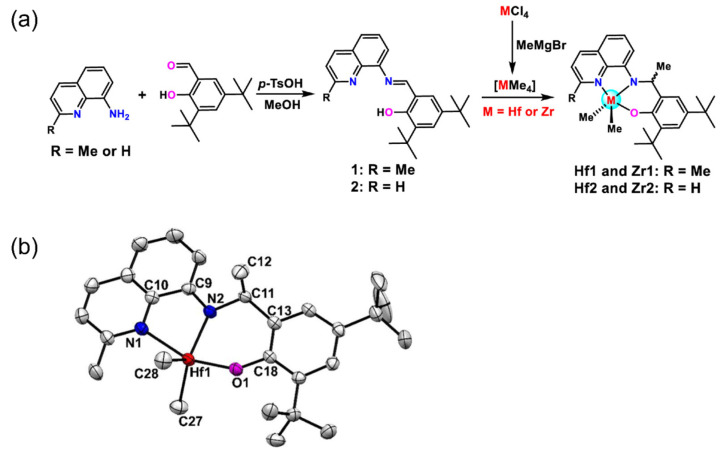
(**a**) Synthesis routes of the quinoline-phenoxyimine complexes; (**b**) Crystal structure of the Hf complex [88]. Reprinted with permission of American chemical society publication, copyright (2025).

Inspired by the high molecular weight capability of quinoline-based *N*,*N*-bidentate ligands under high temperature, the combination of quinoline and phenoxyimine units to construct tridentate ligands offers a promising strategy to improve thermostability. Feng et al. synthesized quinoline-phenoxyimine tridentate ligands via a simple condensation reaction to prepare the corresponding Zr and Hf catalysts (Figure 13a) [88]. Single-crystal X-ray diffraction confirmed the alkyl migration on the imine, which was consistent with *N*,*N*-bidentate imine ligands (Figure 13b). Further polymerization experiments suggested that the Zr complex outperformed the Hf complex, which was consistent with phenoxyimine-based catalysts. The reactive hydrogen atom at the ortho-position of the quinoline nitrogen was prone to attack, leading to the formation of new active centers and broader polymer molecular weight distributions, similar to the quinoline-amino ligands. Additionally, copolymerization of ethylene with 1-octene exhibited a moderate activity of 3.0 × 10^6^ g(polymer)·mol^−1^·h^−1^ and a low insertion rate of 0.51 mol%. The low insertion rates observed for nearly all the tridentate ligands are primarily attributed to the great steric hindrance at the metal center, which inhibits the incorporation of comonomers.

### 4.3. Quinoline-imine-thioether Ligands

**Figure 14:**
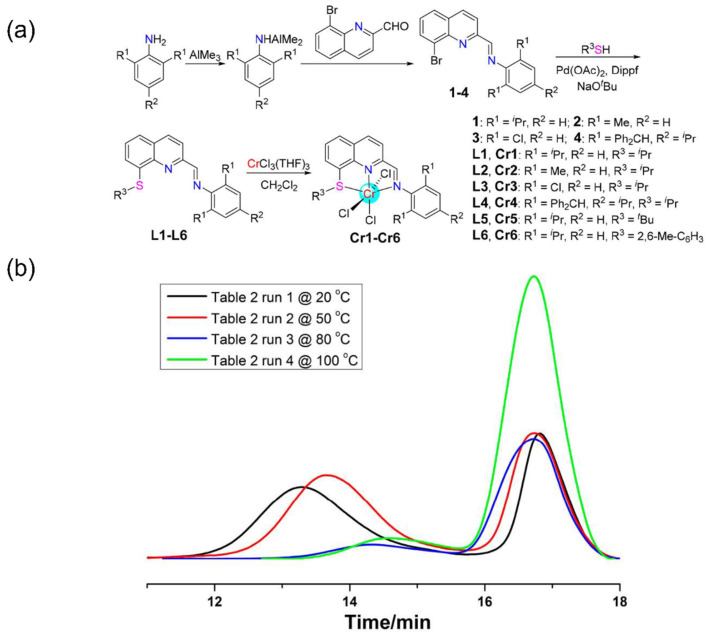
(**a**) Synthesis routes of the quinoline-imine-thioether Cr complexes; (**b**) GPC traces of polyethylenes obtained with Cr1 at different temperatures [89]. Reprinted with permission of American chemical society publication, copyright (2025).

The combination of flexible O, S atoms and the rigid quinoline unit is expected to present diverse catalytic properties. Wang et al. synthesized quinoline-imine-thioether tridentate ligands through imine condensation and Pd-catalyzed coupling of thiols and haloalkanes [89]. To improve the reaction yield, primary amines were first treated with TMA before condensing with quinoline-2-carbaldehyde. Then, the isolated imine was coordinated with CrCl_3_(THF)_3_ to form tridentate Cr complexes (Figure 14a). X-ray single-crystal diffraction confirmed that, unlike quinoline-imine catalysts, the diimine structure retained was attributable to the high migration barrier of chlorine. Ethylene homopolymerization experiments demonstrated that the moderate catalytic activity of these Cr complexes and the resulting polyethylene (PE) exhibited relatively low molecular weights. GPC analysis revealed the presence of two distinct molecular weights over time, indicating the existence of two time-dependent active species during polymerization. Moreover, the active species responsible for high molecular weight gradually deactivated as the temperature increased (Figure 14b). This unique temperature-tunable and time-dependent characteristic makes the Cr complexes suitable for producing bimodal polyethylene, with excellent mechanical strength and superior processability.

Although the quinoline-imine-thioether Cr complex exhibits distinctive characteristics, its catalytic activity is disappointing; this is related to the coordinated metal center. To meet the challenge, Chen et al. synthesized efficient catalysts using the same quinoline-imine-thioether ligand coordinated with Zr and Hf [90]. The corresponding methyl complexes of Zr and Hf were prepared via the classic MMe_4_ method, exhibiting methyl migration as previously observed. The Zr complex exhibited inferior performance to the Hf complex. Polymerization experiments indicated that the Hf complex demonstrated stable activity of 2.2 × 10^7^ g(polymer)·mol^−1^·h^−1^ at 130 °C and 2.5 × 10^7^ g(polymer)·mol^−1^·h^−1^ at 150 °C. However, the high PDI value observed at 150 °C indicated partial decomposition of the Zr/Hf catalysts, with a relatively low comonomer insertion rate of 2.62 mol%.

### 4.4. Pyridine-amino Ligands

Recently, early transition metal tridentate catalysts exhibited some superior properties; however, these systems generally suffered from low comonomer insertion rates when producing POEs. In contrast to the above tridentate catalysts, i.e., imine-amino, imine-enamine, and quinoline-amino catalysts, the pyridine-amino catalysts exhibited significantly better comonomer insertion rate.

**Figure 15:**
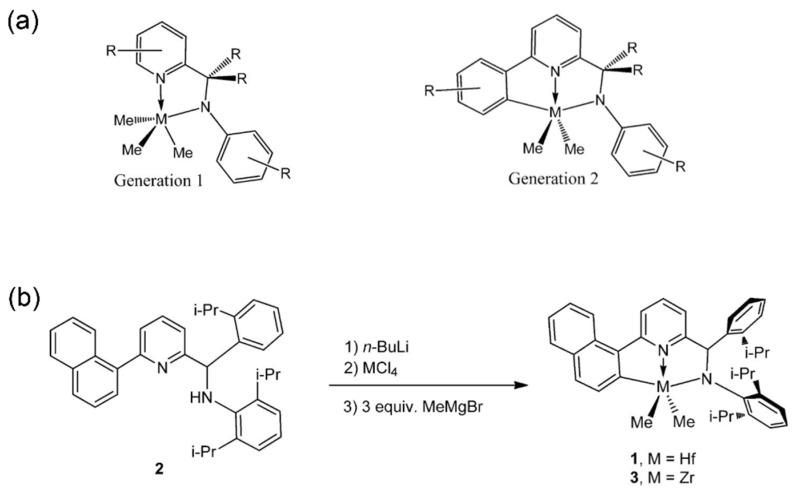
(**a**) Chemical structures of the first- and second-generation pyridine-amino complexes [91]; (**b**) synthesis routes of the naphthalene-bridged pyridine-amino complexes [92]. Reprinted with permission of American chemical society publication, copyright (2025).

The first-generation pyridine-amino catalysts were first developed by Union Carbide based on *N*,*N*-bidentate couplings. Later, Symyx and Dow collaboratively improved the structure, yielding the second-generation tridentate pyridine-amino catalysts (Figure 15a) [91]. These catalysts demonstrated exceptionally high molecular weight capability to produce olefin block copolymers (OBCs), in the presence of chain transfer agents and FI cocatalysts. Subsequently, Dow’s research team developed a naphthalene-bridged metal complex by high-throughput screening technologies (Figure 15b) [92]. As a result, the tridentate pyridine-amino catalyst achieved an extraordinarily high molecular weight capability of 1.4 × 10^6^ and a 1-octene insertion rate of 12.1 mol% at 120 °C, which was unparalleled among the tridentate catalysts. For catalytic activity, the tridentate pyridine-amino catalyst was lower than CGCs and was determined as 1.3 × 10^7^ g(polymer)·mol^−1^·h^−1^.

To further enhance the comonomer insertion efficiency of these pyridine-amino catalysts, Zhang et al. explored the design of bidentate pyridine-amino Hf complexes [93]. Despite the bidentate skeleton, the pyridine-amino catalysts demonstrated remarkable thermal stability of 10^7^ g(polymer)·mol^−1^·h^−1^ at 150 °C. Furthermore, the increased steric accessibility around the metal center facilitated the accommodation of larger comonomers, as evidenced by ethylene/norbornene copolymerization, where an insertion rate of 22.1 mol% was achieved. This high performance underscores its potential for the synthesis of cycloolefin (COC) copolymers. More recently, Yang et al. proposed a steric-tunable tridentate pyridine-amino Hf complex with the introduction of phenolic groups, which effectively minimized steric hindrance and endowed the tridentate complexes with copolymerization capabilities [94]. Consequently, pyridine-amino ligands represent a rare class of tridentate catalysts that simultaneously achieve superior high-temperature activity, molecular weight capability, and comonomer insertion rate.

## 5. Influence of Metal Coordination

The development of non-metallocene catalysts typically follows a two-stage protocol. The initial stage involves ligand synthesis, wherein target ligand compounds are systematically constructed through strategic backbone design with precise modulation of electronic effects and steric hindrance. Subsequently, the coordination reaction between synthesized ligands and metal centers generates diverse non-metallocene catalyst architectures [45,95,96,97,98,99]. While previous sections have extensively addressed the structure–property–application relationships of catalysts derived from various ligand frameworks, it should be emphasized that the metal–ligand coordination process itself critically governs the synthesis optimization pathways, production costs, and ultimate catalytic performance [41,100,101,102,103]. This section provides an evaluation of metal coordination.

The catalytic metals employed in olefin polymerization systems are selected from two distinct groups: Group IVB transition metals (Ti, Zr, Hf) from the early transition series and Group VIII elements (primarily Pd, Ni) representing late transition metals [40,104,105,106,107,108,109,110,111,112]. Late transition metals have garnered significant attention for the copolymerization of olefins with polar monomers, owing to their lower oxophilicity [11,77,113,114,115,116,117,118,119]. These late transition metal catalysts exhibit remarkable versatility in ligand architecture and coordination modes, offering substantial synthetic tunability [120,121,122,123]. However, a critical limitation arises from the predominated coordination bonding that compromises their thermal stability, rendering them unsuitable for high-temperature solution polymerization processes.

The coordination processes involving Group IVB transition metals (Ti, Zr, Hf) are characterized by the partial incorporation of covalent bonding, which significantly enhances the thermal stability of the resulting catalysts [124,125,126,127,128]. Titanium-based systems usually coordinate with metallocene-related ligands, typically through reactions with TiCl_4_ or TiCl_3_(3THF) to form metallocene-Ti(Cl) complexes [129,130,131]. These complexes can subsequently undergo alkylation via Grignard reagents, yielding metallocene-Ti(-CH_3_) derivatives [132,133,134,135]. In contrast, zirconium and hafnium systems present distinct synthetic challenges due to the poor solubility of their chloride precursors. Consequently, more soluble alternatives, such as MBn_4_ or MMe_4_ (M = Zr, Hf), are employed for coordination [136,137,138], while the MMe_4_ route offers near-quantitative conversion with minimal byproduct formation, making it particularly advantageous for laboratory-scale synthesis. However, the MMe_4_ intermediates possess unstable characteristics, requiring stringent cryogenic conditions (<−60 °C) that limit industrial-scale operations. An alternative strategy involves the initial preparation of L-M(ZMe_2_)n followed by its reaction with SiCl_2_Me_2_ to generate the corresponding chlorides [139,140,141]. Comparative analysis reveals that chloride-based catalysts exhibit superior stability and storage characteristics; the trade-off is reduced catalytic activity. Therefore, how to choose needs to be balanced according to the demand.

Another concern is catalyst residuals. These catalysts become irreversibly embedded within the polymer matrix during olefin polymerization, rendering extraction impractical. Their high activity enables catalytic performance at trace concentrations, which generally exert negligible influence on polymer properties while precluding economic viability for reuse [142,143]. However, in photovoltaic-grade POE, even residual metal traces can adversely affect volume resistivity; catalyst residues exert substantially lower impact compared to hundreds or even thousands of times the cocatalyst amount [39,144,145]. Consequently, residual catalysts in polymers are generally not treated further in industry.

## 6. Outlook

This review systematically examined recent advancements in the structural design, catalytic performance, and application landscapes of non-metallocene catalysts. However, it should be noted that most of the current applications remain confined to laboratory-scale demonstrations, with significant technological gaps hindering industrial translation. Critical challenges persist: catalyst storage stability, development of compatible polymerization processes, and optimization of supporting industrial equipment—all of which constitute the primary focus of our research team’s efforts to facilitate commercial adoption. Furthermore, based on the current state of research, some key directions require urgent attention in laboratory-scale catalyst development:

1. **Explore new catalysts:** The growing demand for POE products has led to an increased need for customized catalysts with tailored properties for various application scenarios, while maintaining the basic principles: ease of synthesis, cost-effectiveness, and environmental friendliness. Notably, the development cost of new catalysts can be significantly reduced by integrating virtual screening and machine learning technologies.
2. **Improve quinoline-based catalysts:** The potential of high-thermostability quinolone derivatives has not yet been fully exploited. For further industrial applications, the low insertion rate of quinoline-based tridentate catalysts needs to be addressed immediately.
3. **Clarify structure–performance relationship:** At present, the correlation between catalytic structure and performance remains insufficiently understood. Despite well-recognized electronic effects and steric hindrance capable of tuning the behavior of catalysts, it works only when comparing catalysts of the same types. There is still an absence of a structure–performance basis guiding the development of new catalyst skeletons. The lack of in-depth understanding of the catalytic mechanism constrains the improvement of current catalysts and the design of next-generation catalysts.
4. **Optimize costs:** Further attention must be paid to the fabrication costs of catalysts with applicable value. Currently, most non-metallocene catalysts remain confined to laboratory research, with only a limited number employed in proprietary in-plant applications under confidentiality agreements. The absence of commercial pricing data presents challenges for comprehensive cost–benefit analysis. However, drawing parallels with the commercialization trajectory of metallocene catalysts, excessive production costs would inevitably diminish their market penetration potential. This requires intensive consideration from catalyst design to synthesis route selection and production landing: at the laboratory level, designing streamlined synthesis routes with reduced step counts and simpler reaction conditions, and at the industrial scale, optimizing the reaction and developing purification systems to improve the yield.

## 7. Conclusions

From Ziegler–Natta to metallocene and subsequently to non-metallocene catalysts, the evolution of catalysts has progressed from multisite to single-site active centers, from water- and oxygen-sensitive to environmentally tolerant, and from thermal deactivation to thermostability. At present, although non-metallocene catalysts are currently not widely applied in industry, their unique characteristics are noteworthy for the production of POEs, such as high-temperature molecular weight retention and simple synthesis routes. According to the data of each type of non-metallocene catalyst in Table 2, the key characteristics (including structural features, advantages and disadvantages, and stability) and appropriate applications of each catalyst are summarized here:

(a) *N*,*N*-Bidentate catalysts (including imine-amino, imine-enamine, and quinoline-imine) are derived from diimine catalysts. In these complexes, an imine nitrogen atom participates in the metal center via coordination interactions, while the other nitrogen forms a covalent bond with metal. The incorporation of covalent bonding significantly enhances thermal stability. And the relatively open coordination geometry facilitates copolymer monomer insertion, endowing *N*,*N*-bidentate catalysts with performance metrics comparable to metallocene catalysts (imine-enamine). Notably, the synthesis conditions required for ligand preparation confer advantages in terms of industrial scale-up and cost-effectiveness, contrasting sharply with the strict anhydrous/oxygen-free environments typically mandated for metallocene ligand synthesis. Among these catalysts, quinoline-imine, although compromised in catalytic activity, deserves further attention as its high-temperature-resistant features allow higher efficiency of mass and heat transfer in industrial production.
(b) *N*,*O/S*-Bidentate ligands, structurally characterized by phenoxy-imine or thioether-amine motifs, originate from FI catalyst derivatives. These systems generally display suboptimal thermal stability, catalytic activity, and copolymer monomer incorporation capabilities. Consequently, they are predominantly employed in combination with other catalysts for the production of OBC.
(c) Tridentate ligands typically combine *N*,*N*-bidentate and *N*,*O/S*-bidentate, creating sterically crowded metal centers that severely restrict copolymer monomer insertion. This structural limitation confines their primary application to LLDPE production. A special case is the pyridine-amino type of catalysts, which achieve copolymerization capabilities equivalent to imine-enamine catalysts. Despite exhibiting polymerization activity one order of magnitude lower than imine-enamine systems, their unique synergy with FI catalysts in OBC manufacturing has attracted significant research attention.

While non-metallocene catalysts currently lag behind metallocene catalysts in copolymerization capability and molecular weight regulation, they exhibit comparable activity alongside two critical advantages: enhanced thermal stability and simplified ligand synthesis protocols. These combined attributes improve feasibility and are cost-effective for industrial manufacturing. Furthermore, catalysts based on imino-enamine and pyridine-imine units exhibit comparable performance to CGCs, offering significant improvement.

Despite the inherent advantages of non-metallocene catalysts, only limited variants (e.g., pyridine-imine and O4-type catalysts) have achieved commercial viability to date. Challenges remain in establishing an effective catalyst backbone (currently highly dependent on high-throughput screening techniques), simplifying synthesis routes and improving profitability. In addition, the market landscape is further complicated by impending oversaturation in downstream polyolefin elastomer (POE) applications, compressing the profitability in the future. However, emerging opportunities lie in the growing demand for differentiated polymer properties. How to find new products that better appeal to the market would be a potential growth point in the future. In conclusion, research in the field of non-metallocene catalysts and their industrialization is full of opportunities and challenges.

## Figures and Tables

**Table 1:** Copolymerization data of ethylene/1-octene polyolefin elastomers at 120 °C ^a^.

Procatalyst	Octene Incorp (wt%)	Polymer (g)	Activity (g poly/mmol cat.)	*M*_W_ (g/mol)	*M*_n_ (g/mol)	Polymer Dispersity Index	*T*_C_ (°C)
Imino-amido	9.8	18.3	24.339	390.690	124.180	3.27	108
Imino-enamido	18.3	23.5	31.352	640.130	145.290	4.16	87
6a	13.4	19.2	25.599	280.300	100.500	2.79	95
6b	16.5	30.9	41.165	403.610	116.760	3.46	89
6c	6.9	10.6	14.094	159.800	54.580	2.93	122
6d	14.9	28.3	37.715	632.200	212.500	2.97	93

^a^ Reactor size = 1 gal. Ethylene pressure = 425 psi (145 g of ethylene). The reactor was also charged with 250 g of 1-octene, 1350 g of Isopar E, and 20 mmol of H_2_ and heated to 120 °C. Then, 0.75 μmol of each catalyst was injected along with 0.85 μmol of cocatalyst and 22.5 μmol of modified methylaluminoxane. Run time was 10 min. All catalysts were inactive after 10 min run time under these conditions, as evidenced by ethylene uptake monitoring [66]. Reprinted with permission of American chemical society publication, copyright (2025).

**Table 2:** Data of the concluded catalysts (optional candidate for ethylene/1-octene copolymerization at 120 °C).

Catalysts	Route Numbers	Yield (%)	Activity (g/mmolcat.) ^a^	Incorp Rate (wt%) ^b^	Advantage	Application
Imino-amido [59]	5	77	140,800	3.8/14.9	High molecular weight	POE POE POE
Imino-enamido [61]	6	57	195,500	7.0/14.8	High activity, Mw, and incorp rate	
Amido-quinoline [66]	4	85	55,417	13.3/25.3	Thermally stable	
Phenoxy-imine-amino [87]	5	85	67,500	0.29	Multi-coordinable metal	LLDPE LLDPE LLDPE
Phenoxy-imine-quinoline [88]	3	77	79,200 (100 °C)	0.64	Facile synthesis	
Quinoline-imine-thioether [90]	4	79	22,110 (130 °C)	1.7	Thermally stable	
Pyridine-amino [92]	6	78	13,267	12.1/15.6	High incorp rate Product OBC	POE, OBC

^a^ The closest temperature is used instead of 120 °C when data are not available; ^b^ The latter data represent the incorporation rates of the CGC under the corresponding conditions, thus making the data directly comparable (different experimental parameters have a strong influence on the incorporation rates).

## Data Availability

The original contributions presented in this study are included in the article. Further inquiries can be directed to the corresponding authors.

## Abbreviations

The following abbreviations are used in this manuscript:

POE	Polyolefin elastomer
Z-N	Ziegler–Natta
MAO	Methylaluminoxane
Cp_2_ZrCl_2_	dichlorodicyclopentadienyl zirconium
CGC	Constrained geometry catalyst
Cp	Cyclopentadienyl
EPOE	Ethylene-based polyolefin elastomer
OBC	Olefin block copolymer
PE	Polymer dispersity index
^i^Pr	Isopropyl
Me	Methyl
PE	Polyethylene
FI	Phenoxy-imine
O_4_	Four-oxygen coordination structure
M	Metal
LLDPE	Linear low-density polyethylene
THF	Tetrahydrofuran
GPC	Chromatography
TMA	Trimethylaluminum
COC	Copolymers of cycloolefin
